# CXCL11 production in cerebrospinal fluid distinguishes herpes simplex meningitis from herpes simplex encephalitis

**DOI:** 10.1186/s12974-017-0907-5

**Published:** 2017-07-10

**Authors:** Liza Lind, Marie Studahl, Linn Persson Berg, Kristina Eriksson

**Affiliations:** 1 Department of Rheumatology and Inflammation Research, Sahlgrenska Academy, University of Gothenburg, Gothenburg, Sweden; 2 Department of Infectious Diseases, Sahlgrenska Academy, University of Gothenburg, Gothenburg, Sweden

**Keywords:** Herpes simplex, Encephalitis, Meningitis, Chemokine, Cytokine

## Abstract

**Background:**

The closely related herpes simplex viruses 1 and 2 can cause inflammations of the central nervous system (CNS), where type 1 most often manifest as encephalitis (HSE), and type 2 as meningitis (HSM). HSE is associated with severe neurological complications, while HSM is benign in adults. We proposed that studying the chemokine and cytokine production in cerebrospinal fluid (CSF) and serum could indicate why two closely related viruses exhibit different severity of their accompanied CNS inflammation.

**Methods:**

Secretion patterns of 30 chemokines and 10 cytokines in CSF of adult patients with acute HSE (*n* = 14) and HSM (*n* = 20) in the initial stage of disease were analyzed and compared to control subjects without viral central nervous system infections and to levels in serum.

**Results:**

Most measured chemokines and cytokines increased in CSF of HSE and HSM patients. Overall, the CSF chemokine levels were higher in CSF of HSM patients compared to HSE patients. However, only five chemokines reached levels in the CSF that exceeded those in serum facilitating a positive CSF-serum chemokine gradient. Of these, CXCL8, CXCL9, and CXCL10 were present at high levels both in HSE and HSM whereas CXCL11 and CCL8 were present in HSM alone. Several chemokines were also elevated in serum of HSE patients but only one in HSM patients. No chemokine in- or efflux between CSF and serum was indicated as the levels of chemokines in CSF and serum did not correlate.

**Conclusions:**

We show that HSM is associated with a stronger and more diverse inflammatory response in the CNS compared to HSE in the initial stage of disease. The chemokine patterns were distinguished by the exclusive local CNS production of CXCL11 and CCL8 in HSM. Inflammation in HSM appears to be restricted to the CNS whereas HSE also was associated with systemic inflammation.

**Electronic supplementary material:**

The online version of this article (doi:10.1186/s12974-017-0907-5) contains supplementary material, which is available to authorized users.

## Background

Herpes simplex viruses (HSV) may cause inflammations in the central nervous system (CNS), manifesting either as encephalitis or meningitis. Encephalitis is most often caused by herpes simplex virus type 1 (HSV-1), whereas meningitis primarily is caused by HSV type 2 (HSV-2) [1]. HSV-1 encephalitis (HSE) occurs with an incidence of 2–4/million, and the estimate for HSV-2 meningitis (HSM) is 2–4/100,000 [1–3]. Women are six times more likely to develop HSM compared to men, whereas no sex predilection is found for HSE [1, 4, 5].

HSV-1 and HSV-2 are double-stranded DNA viruses with approximately 50% DNA homology. Both viruses initially infect mucosal tissue and later establish latent infections in the CNS [6]. HSV-1 mainly cause cold sores and propagate to the CNS from the trigeminal and/or olfactory ganglia to the temporal lobe. From there the virus may spread to the contralateral temporal lobe possibly via the anterior commissure [7]. The temporal inflammatory lesions on magnetic resonance imaging (MRI) seen in the majority of HSE patients give rise to symptoms with high fever, headache, seizures, disorientation, and dysphasia [8]. HSE is associated with high mortality without antiviral treatment (>70%), and severe neurological sequelae may appear regardless of antiviral treatment [9, 10]. HSV-2 commonly infects through the genital mucosa and spreads to the CNS where it is thought to emanate from the sacral ganglia. HSM symptoms are more benign with fever, headache, stiff neck, and photosensitivity, even though some patients develop more severe symptoms such as sacral radiculomyelitis [11]. HSM, in contrast to HSE, is characterized by recurrent episodes of disease [2, 11, 12]. The CSF inflammatory response in HSE and HSM is characterized by pleocytosis, mainly lymphocytes, and increased protein content [5], where the latter generates a more pronounced pleocytosis [1, 11]. During HSE, there is a vigorous intrathecal innate immune response necessary for controlling the viral infection including inflammatory markers such as IL-6 and TNF-α [13], but this response may concurrently contribute to brain tissue destruction and consequent neurologic sequelae. HSM is associated with induction of alfa- and gamma interferon (IFN), but the immune response is generally less well characterized [14, 15]. Both diseases are treated with acyclovir, a selective inhibitor of viral replication that potently reduces viral titers and lowers mortality in HSE [16–18].

Leukocyte entry into the CNS is restricted by the blood-brain barrier (BBB) and/or the blood-cerebrospinal fluid barrier (BCSFB); hence, the CNS is considered immune privileged. However, immune surveillance continuously takes place in the CNS even in non-inflammatory states, suggesting controlled mechanisms of leukocyte trafficking over the barriers and into the cerebrospinal fluid (CSF) [19–22]. Inflammatory cells migrate to peripheral tissues in response to chemokines, i.e., chemotactic cytokines. Chemokine signaling occurs through G protein-coupled chemokine receptors which are present on all immune cells [23]. The expression profile of chemokine receptors differ between different leukocyte subsets, between cells in different tissues, as well as between resting and activated cells, and determine the migratory potential and homing pattern of all white blood cells [24]. The homeostatic secretion profiles of chemokines and the expression pattern of their respective receptors in the CNS of humans is relatively unexplored [23], as is the characterization of inducible inflammatory chemokines during viral CNS infections. Best described are CXCL9 and CXCL10 which both bind CXCR3, a chemokine receptor primarily expressed on T cells and natural killer (NK) cells [25, 26]. CXCR3 also binds the less well-characterized CXCL11. All three CXCR3 ligands are upregulated in response to IFN-γ, a potent pro-inflammatory cytokine [27].

To identify parameters associated with the recruitment of immune cells to the CNS in HSE and HSM, we conducted analysis of 30 chemokines and 10 cytokines in the CSF of adult patients with HSE or HSM and compared to control subjects without viral CNS infections. To analyze potential in- and/or efflux of chemokines/cytokines between CSF and serum, we compared paired CSF and serum samples from these patients. We found that most measured chemokines and cytokines increased in the CSF of both HSE and HSM patients. However, immune cells move toward a chemokine gradient, which imply that levels in CSF need to exceed those in serum for potent cell migration into the CNS. This criterion was filled for CXCL8, CXCL9, and CXCL10 in both HSE and HSM. Two chemokines distinguished HSE from HSM; CCL8 and in particular CXCL11 was only expressed above serum levels in the CSF of HSM but not HSE patients. We thus conclude that HSM, despite being a more benign disease, is characterized by a stronger and a more diverse inflammatory CSF chemokine response.

## Methods

### Study population and procedures

Patients with suspected HSE or HSM were admitted to the Department of Infectious Diseases, Sahlgrenska University Hospital, Gothenburg, Sweden. Patients with HSE had clinical signs of encephalitis with fever, disorientation, altered consciousness, paresis, seizures and/or dysphasia, and MR or CT-changes compatible with herpes encephalitis. Patients with HSM had clinical signs of meningitis with headache, nausea and/or vomiting, and pleocytosis in lumbar puncture. HSE and HSM, respectively, were confirmed on admission to the hospital by detection of HSV-1 or HSV-2 DNA by quantitative in-house TaqMan PCR methods [28]. Cerebrospinal fluid samples from 14 patients with confirmed HSE and 20 patients with confirmed HSM were included in this study. Persons that sought care for headaches, but did not have pleocytosis and confirmed negatively for bacteria and virus in the CSF were used as controls (*n* = 35). None of the control patients were diagnosed with any neurologic condition including infection or autoimmunity during a 1-year follow-up period. As none of the patients had pleocytosis, we find it unlikely that they had an undiagnosed CNS infection. The cerebrospinal fluid samples were centrifuged 1500 rpm for 5 min to remove cells. Eight patients with HSE and 8 patients with HSM had paired CSF and serum samples (±2 days). In the control group, 3 persons had paired CSF and serum, while CSF was obtained from 30 persons, and additional sera from 2 persons. All HSE and HSM samples were obtained during acute infection within 20 days after symptom onset and stored in −70 °C. The medical records were obtained for registration of clinical data including laboratory results on CSF albumin levels, pleocytosis, and treatment.

Patients with HSE (8 women, 6 men) had the median age of 64.5 years (range 27–89 years). Patients were treated with acyclovir 10–15 mg/kg 3 times daily intravenously for 2–3 weeks. Patients with HSM (17 women, 3 men) had the median age of 42.5 years (range 31–64 years). Treatment was given after lumbar puncture and serum sampling with oral valaciclovir 1 g 3 times daily for 1 week, initially replaced by intravenously acyclovir 5 mg/kg 3 times daily in case of difficulties with oral administration. Control subjects (17 women, 18 men) had the median age of 53 years (range 21–91 years). Routine clinical analysis data of albumin levels and number of mono- and polynuclear cells in CSF were retrieved from medical records of HSE and HSM patients. Reference values for healthy individuals are for albumin <320 mg/L (15–45 years) and <420 mg/L (>45 years), for mononuclear cells <5 × 10^6^ cells/L and for polynuclear cells <3 × 10^6^ cells/L [29].

### Analysis of cytokines and chemokines

Thirty chemokines and 10 cytokines (CCL1, CCL2, CCL3, CCL7, CCL8, CCL11, CCL13, CCL15, CCL17, CCL19, CCL20, CCL21, CCL22, CCL23, CCL24, CCL25, CCL26, CCL27, CX3CL1, CXCL1, CXCL2, CXCL5, CXCL6, CXCL8, CXCL9, CXCL10, CXCL11, CXCL12, CXCL13, CXCL16, GM-CSF, IFN-γ, IL-1β, IL-2, IL-4, IL-6, IL-10, IL-16, MIF, and TNF-α) were detected and quantified in duplicate samples of serum or CSF using Bio-Plex Pro™ Assay (Bio-Rad Laboratories) according to the manufacturer’s instructions. Briefly, samples and standards were mixed with capture beads, were incubated in the dark in room temperature (RT) for 1 h on a shaker at 850 rpm, and were subsequently washed. Biotin-labeled antibodies were added to the capture beads and incubated for 30 min as described above. Following the washing step capture bead/biotin antibody complexes were incubated with Streptavidin-PE for 10 min. Complexes were washed and measured by a Bio-Plex 200 System (Bio-Rad Laboratories) with 5-parameter logistics standard curves which were used for interpolation of chemokine and cytokine levels.

### Statistics

Normal distribution was tested using Shapiro-Wilk normality test. Albumin levels and mono- and polynuclear cell numbers were compared using Mann-Whitney *U* test. For multiple comparisons of the three groups (HSE and HSM patients and control subjects) Kruskal-Wallis’ non-parametric test with Dunn’s post-test was used (*n* = 8–33/group). Statistical differences between serum and CSF in patient groups were calculated using Wilcoxon matched-pairs signed rank test (*n* = 8/group). Non-parametric Spearman’s correlation coefficient was used to calculate correlation between serum and CSF (*n* = 8/group). All statistical analysis was performed using GraphPad Prism version 6 (GraphPad Software).

## Results

### HSM is associated with higher levels of mononuclear cells compared to HSE

The levels of albumin and the number of mono- and polynuclear cells in CSF were measured during routine clinical analysis and are summarized in Table 1. As previously described, HSM were accompanied by a significantly higher influx of mononuclear cells in the CSF, compared to HSE. No significant difference between the two patient groups was observed in either levels of albumin or number of polynuclear cells.

**Table 1:** Albumin, mono-, and polynuclear cells in CSF of HSE and HSM patients

		HSE	HSM	*p*
CSF albumin (mg/L)^a^	Median (range)	617 (215–1010)	762 (345–2385)	0.072
CSF monunuclear cells (×10^6^/L)	Median (range)	118.5 (22–272)	206.5 (69–916)	0.0064
CSF polynuclear cells (×10^6^/L)	Median (range)	2.5 (0–34)	5 (1–57)	0.24

^a^ For one patient with HSE and three patients with HSM CSF albumin data could not be obtained

*Abbreviations: HSE* herpes simplex encephalitis, *HSM* Herpes simplex meningitis, *CSF* Cerebrospinal fluid

### In health CCL2, CXCL8, CXCL10, CXCL12, and CXCL16 are expressed at higher levels in CSF compared to serum

**Fig. 1:**
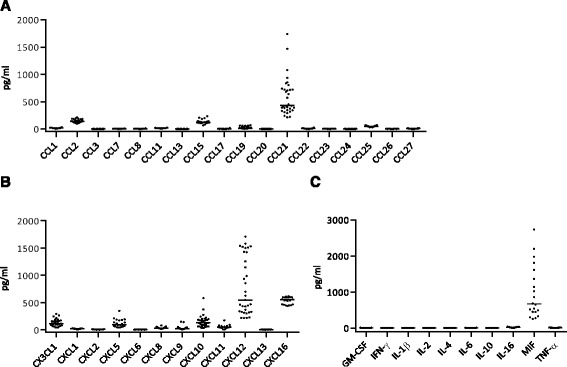
Levels of **a** CC chemokines, **b** CXC chemokines, including CX3CL1, and **c** cytokines in the CSF of healthy control subjects (*n* = 20–33). Data are presented as individual values with medians indicated by *horizontal bars*

Baseline levels of cytokines and chemokines were examined in CSF and serum of control subjects without viral CNS infection (*n* = 5–33). In the CC chemokine group five chemokines (CCL2, CCL15, CCL19, CCL21, CCL25) were constitutively expressed in CSF (Fig. 1a, Table 2), but only CCL2 had higher median expression in CSF (Table 2) compared to serum (Additional file 1: Table S1). In the CX chemokine group (including CX3CR1), eight measured chemokines (CX3CL1, CXCL5, CXCL8, CXCL9, CXCL10, CXCL11, CXCL12, CXCL16) were constitutively expressed in CSF (Fig. 1b, Table 2). Among these chemokines, four had higher median expression in CSF (Table 2) compared to serum (Additional file 1: Table S1), namely, CXCL8, CXCL10, CXCL12, and CXCL16. The remaining chemokines were expressed at negligible levels in CSF (Fig. 1a–b, Table 2). None of the ten cytokines analyzed were expressed during steady-state in CSF except for the chemokine-like cytokine macrophage migration inhibitory factor (MIF) (Fig. 1c, Table 2).

**Table 2:** Levels of chemokines and cytokines in cerebrospinal fluid of HSE and HSM patients and healthy controls

	CSF-median and range (pg/ml)						*p* HSE vs. control	*p* HSM vs. control	*p* HSE vs. HSM
Cytokine	HSE		HSM		Control				
CCL1	31	5–141	21	15–29	17	3–34	0.0032	0.41	0.63
CCL2	186	23–1179	233	41–1565	141	101–215	>0.99	0.19	0.62
CCL3	10	2–15	12	9–27	1	1–2	<0.0001	0.0001	>0.99
CCL7	13	3–42	35	16–57	4	1–8	0.0020	<0.0001	0.27
CCL8	63	4–560	1085	117–8734	5	2–9	0.0012	<0.0001	0.26
CCL11	20	5–41	26	16–36	16	5–22	0.012	0.0007	0.87
CCL13	3	1–15	14	7–105	1	0–2	0.094	<0.0001	0.041
CCL15	264	87–673	378	230–710	123	66–232	0.011	0.0003	0.61
CCL17	21	0–83	15	11–42	2	0–20	0.0001	0.0015	>0.99
CCL19	104	7–339	97	9–259	16	3–69	<0.0001	<0.0001	>0.99
CCL20	1	0–3	3	0–14	0	0–1	0.014	<0.0001	0.50
CCL21	234	58–1369	341	17–822	431	214–1745	0.011	0.025	>0.99
CCL22	34	3–123	38	16–74	9	0–27	0.0017	0.0009	>0.99
CCL23	20	3–126	14	10–27	4	2–10	<0.0001	0.0009	>0.99
CCL24	7	0–19	10	4–17	0	0–6	0.0069	0.0001	0.59
CCL25	91	38–174	139	104–199	51	29–72	0.0019	<0.0001	0.27
CCL26	4	1–16	7	3–10	3	1–5	0.43	0.0024	0.17
CCL27	5	2–11	8	4–16	6	1–13	>0.99	0.21	0.22
CX3CL1	183	71–296	186	38–1016	107	38–289	0.018	0.0011	>0.99
CXCL1	42	21–669	101	9–837	16	5–24	<0.0001	<0.0001	>0.99
CXCL2	7	2–17	11	5–16	4	0–8	0.018	<0.0001	0.32
CXCL5	179	58–461	334	204–708	91	41–346	0.065	0.0002	0.23
CXCL6	4	0–17	8	4–91	0	0–1	0.0004	<0.0001	0.56
CXCL8	102	21–3450	759	24–7130	27	11–77	0.0006	<0.0001	0.30
CXCL9	1877	22–8429	2711	9–11035	19	5–146	<0.0001	<0.0001	>0.99
CXCL10	4508	78–19596	6905	108–27737	125	30–578	<0.0001	<0.0001	>0.99
CXCL11	250	10–3220	6789	2214–9424	42	4–171	0.034	<0.0001	0.047
CXCL12	740	342–1414	423	64–1839	542	213–1708	>0.99	0.80	0.35
CXCL13	16	2–34	5	1–26	1	0–4	<0.0001	<0.0001	0.21
CXCL16	453	326–748	357	159–395	548	437–615	0.32	<0.0001	0.027
GM-CSF	12	0–16	25	10–34	1	0–10	0.037	<0.0001	0.10
IFN-γ	1	0–8	8	4–19	0	0–1	0.13	<0.0001	0.13
IL-1β	3	1–5	7	3–12	1	1–2	0.0025	<0.0001	0.17
IL-2	2	0–3	15	5–29	0	0–1	0.0020	<0.0001	0.12
IL-4	2	1–5	3	1–3	2	0–7	>0.99	>0.99	0.89
IL-6	133	4–2629	1192	123–3627	3	2–11	0.0006	<0.0001	0.35
IL-10	17	4–47	46	16–129	4	2–7	0.0012	<0.0001	0.31
IL-16	73	15–325	156	77–233	24	6–39	0.014	0.0001	0.39
MIF	1303	129–17013	4675	794–7144	666	254–2733	0.35	0.0009	0.12
TNF-α	29	7–74	48	28–63	9	2–16	0.0006	<0.0001	0.52

### CXCL8, CXCL9, and CXCL10 are markedly increased in CSF of HSE and HSM patients

CSF samples from patients with HSE or HSM were analyzed for chemokines and cytokines (*n* = 8–20). 28 out of 30 chemokines increased significantly in CSF of HSE and/or HSM patients compared to the levels in control patients without CNS infections (Table 2). However, cells are believed to migrate from blood into the CNS by a chemokine gradient. We therefore focused on chemokines that reached levels in the CSF exceeding those in serum (i.e., a CSF/serum factor >1 and a significant difference between the two parameters) (Table 3) and argue that these are of biological significance for cell migration to the CNS.

**Table 3:** Comparison of chemokines and cytokines, including correlations, in CSF and serum of HSE and HSM patients

	Factor CSF/serum		*p* CSF vs. serum		Correlation HSE		Correlation HSM	
Cytokine	HSE	HSM	HSE	HSM	*r*	*p*	*r*	*p*
CCL1	0.5	0.4	0.016	0.0078	0.3	0.50	0.4	0.30
CCL2	3	5	0.016	0.0078	−0.5	0.20	−0.4	0.36
CCL3	1	2	0.15	0.0078	0.2	0.66	0.4	0.39
CCL7	0.2	0.7	0.023	0.055	−0.6	0.15	0.8	0.015
CCL8	0.7	23	0.74	0.0078	−0.2	0.66	−0.1	0.75
CCL11	0.1	0.2	0.0078	0.0078	0.3	0.50	0.3	0.50
CCL13	0.03	0.2	0.0078	0.039	−0.1	0.84	0.1	0.88
CCL15	0.05	0.1	0.0078	0.0078	0.5	0.27	0.1	0.79
CCL17	0.1	0.2	0.0078	0.0078	0.2	0.66	−0.1	0.79
CCL19	0.3	0.4	0.039	0.11	−0.1	0.84	0.7	0.069
CCL20	0.07	0.5	0.0078	0.023	0.0	0.93	0.7	0.070
CCL21	0.1	0.2	0.0078	0.0078	0.4	0.30	0.3	0.43
CCL22	0.09	0.05	0.0078	0.0078	0.1	0.79	0.2	0.66
CCL23	0.05	0.04	0.0078	0.0078	0.2	0.70	−0.1	0.87
CCL24	0.03	0.04	0.0078	0.0078	0.5	0.24	0.8	0.028
CCL25	0.1	0.3	0.0078	0.0078	−0.3	0.54	0.3	0.54
CCL26	0.2	0.3	0.0078	0.0078	0.2	0.70	0.5	0.24
CCL27	0.01	0.01	0.0078	0.0078	0.0	0.93	0.1	0.88
CX3CL1	0.7	0.8	0.016	0.15	0.1	0.75	0.5	0.22
CXCL1	0.2	0.6	0.31	0.25	−0.5	0.20	0.3	0.50
CXCL2	0.01	0.03	0.0078	0.0078	−0.3	0.46	0.5	0.26
CXCL5	0.1	0.4	0.0078	0.016	−0.1	0.75	0.2	0.70
CXCL6	0.1	0.2	0.0078	0.11	−0.8	0.022	0.5	0.20
CXCL8	3	40	0.0078	0.0078	−0.5	0.27	0.6	0.12
CXCL9	4	12	0.023	0.0078	0.7	0.046	−0.02	0.98
CXCL10	64	66	0.0078	0.0078	0.5	0.27	−0.05	0.93
CXCL11	0.4	31	>0.99	0.0078	0.1	0.75	0.1	0.79
CXCL12	0.9	0.6	0.84	0.055	0.5	0.24	−0.1	0.75
CXCL13	0.7	0.2	0.055	0.0078	0.8	0.037	0.1	0.79
CXCL16	0.7	0.7	0.20	0.023	−0.9	0.011	0.6	0.15
GM-CSF	n/a	n/a	0.039	0.55	0.2	0.66	0.5	0.17
IFN-γ	n/a	n/a	0.38	0.0078	−0.6	0.10	−0.1	0.84
IL-1β	n/a	n/a	0.0078	0.0078	−0.4	0.33	0.6	0.13
IL-2	n/a	n/a	0.023	0.016	−0.6	0.10	−0.01	0.99
IL-4	n/a	n/a	0.0078	0.0078	−0.6	0.12	0.5	0.27
IL-6	n/a	n/a	0.078	0.0078	−0.7	0.058	0.4	0.28
IL-10	n/a	n/a	0.055	0.64	−0.7	0.046	−0.1	0.88
IL-16	n/a	n/a	0.31	0.15	−0.7	0.046	−0.1	0.79
MIF	n/a	n/a	0.25	0.016	0.2	0.66	−0.1	0.84
TNF-α	n/a	n/a	0.55	0.0078	−0.5	0.24	0.6	0.15

**Fig. 2:**
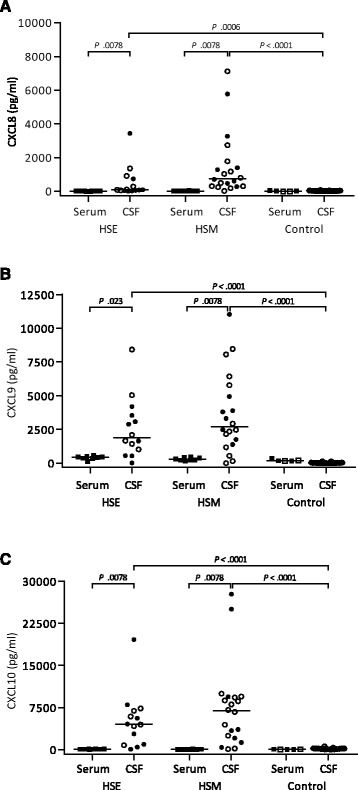
Levels of **a** CXCL8, **b** CXCL9, and **c** CXCL10 in CSF of HSE (*n* = 14) and HSM (*n* = 20) patients compared to healthy controls (*n* = 33) and to serum (*n* = 8/group; control = 5). Data are presented as individual values with medians indicated by *horizontal bars*. CSF comparisons were performed using Kruskal-Wallis’ non-parametric test with Dunn’s post-test. *White symbols* are non-matched CSF or serum samples, which were excluded from analysis of paired CSF and serum (*filled symbols*), using Wilcoxon matched-pairs signed-rank test. Statistical analysis was performed using GraphPad Prism version 6 (GraphPad Software). Abbreviations: *CSF* cerebrospinal fluid, *HSE* herpes simplex encephalitis, *HSM* herpes simplex meningitis

CXCL8, CXCL9, and CXCL10 increased in the CSF of HSE and HSM patients and reached levels in the nanogram/ml range (Fig. 2a–c). The induction of CXCL8 and CXCL9 were highly significant in HSM (*p* = <0.0001) as well as HSE (*p* = 0.0006 and *p* = <0.0001, respectively) (Fig. 2a–b, Table 2), but the CSF-serum gradient was much more pronounced in HSM (factor of 40 and 12, respectively) compared to the HSE patient group (factor of 2.6 and 3.8, respectively) (Table 3). There was a remarkable increase of CXCL10 in both HSE and HSM (*p* = <0.0001) (Fig. 2c) with a CSF-serum factor of around 65 for both patient groups (Table 3).

### High levels of CXCL11 and CCL8 in CSF are associated with HSM but not HSE

**Fig. 3:**
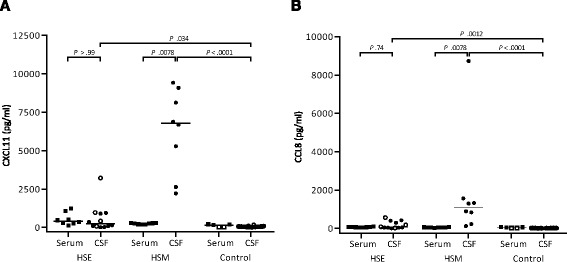
Levels of **a** CXCL11 and **b** CCL8 in CSF of HSE (*n* = 12) and HSM (*n* = 8) patients compared to healthy controls (*n* = 20) and to serum (*n* = 8/group; control = 5). Data are presented as individual values with medians indicated by *horizontal bars*. CSF comparisons were performed using Kruskal-Wallis’ non-parametric test with Dunn’s post-test. *White symbols* are non-matched CSF or serum samples, which were excluded from analysis of paired CSF and serum (*filled symbols*), using Wilcoxon matched-pairs signed rank test. Statistical analysis was performed using GraphPad Prism version 6 (GraphPad Software). Abbreviations: *CSF* cerebrospinal fluid, *HSE* herpes simplex encephalitis, *HSM* herpes simplex meningitis

Among 30 measured chemokines, 2 distinguished HSE from HSM. The most impressive response was observed for CXCL11 which increased enormously in HSM (*p* = <0.0001) (Fig. 3a, Table 2). CXCL11 reached levels between 2 and 10 ng/ml in all HSM patients but remained low in most HSE patients (Fig. 3a), with a CSF-serum factor of 31 for HSM, compared to 0.35 in HSE (Table 3). CCL8 had a median >200-fold increase in CSF of HSM patients, compared to 13 for the HSE group (Fig. 3b, Table 2). However, only HSM was associated with a positive CSF-serum gradient (Table 3). To rule out that the differences in CSF levels of CXCL11 and CCL8 in HSE and HSM patients were not due to gender bias (the predominance of females in the HSM cohort), we performed a subgroup analysis of females only which shows that these chemokines are indeed higher in HSM irrespective of gender (Additional file 2: Figure S1). We could not perform a similar statistical evaluation for males as they were too few in the HSM cohort.

Of the remaining measured chemokines most increased in CSF of both HSM and HSE, excluding only CCL2, CCL27, and CXCL12, but the levels were overall modest (Table 2), and all with a CSF/serum factor < 1 (Table 3). In serum, six chemokines were elevated in HSE (CCL20, CCL27, CX3CL1, CXCL2, CXCL9, and CXCL11) compared to only one in HSM (CCL21) (Additional file 1: Table S1).

### Proinflammatory cytokines are induced in CSF of HSE and HSM patients

**Fig. 4:**
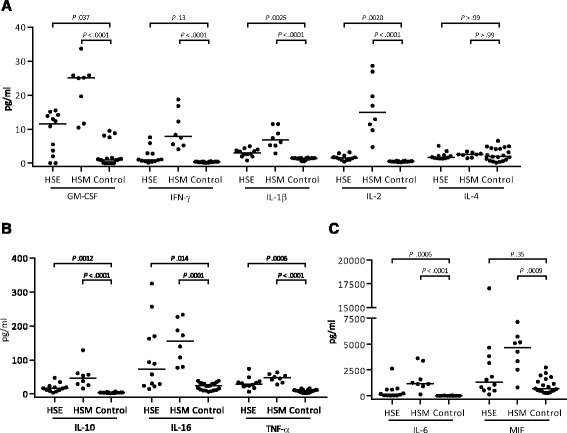
Levels of cytokines in CSF of HSE (*n* = 12) and HSM (*n* = 8) patients compared to healthy controls (*n* = 20–33). **a** GM-CSF, IFN-γ, IL-1β, IL-2, and IL-4. **b** IL-10, IL-16, and TNF-α. **c** IL-6 and MIF. Data are presented as individual values with medians indicated by *horizontal bars*. CSF comparisons were performed using Kruskal-Wallis’ non-parametric test with Dunn’s post-test in GraphPad Prism version 6 (GraphPad Software). Abbreviations: *CSF* cerebrospinal fluid, *HSE* herpes simplex encephalitis, *HSM* herpes simplex meningitis

Most cytokines analyzed were induced in CSF of both HSM and HSE patients (Table 2). IFN-γ, the hallmark of an anti-viral Th1 response, increased significantly for HSM, but not HSE (Fig. 4a). GM-CSF, IL-1β, and IL-2 increased in both patient groups (Fig. 4a). IL-4, which is most commonly associated with Th2-responses, increased neither in HSE nor in HSM (Fig. 4a). IL-10, IL-16, and TNF-α had a pronounced actual increase in both HSE and HSM (Fig. 4b), although the increase was not significant for IL-16 in HSE (Table 2). IL-6 and MIF were the dominant cytokines reaching concentrations in the nanogram range (Fig. 4c), but again the increase for MIF in HSE was not significant (Table 2). The only cytokines that increased in serum was MIF (*p* = 0.02, Additional file 1: Table S1), and only in HSM patients.

### Chemokine and cytokine levels in CSF of HSE and HSM patients are not reflected in the systemic compartment

To evaluate the potential in- or efflux of chemokines and/or cytokines between serum and CSF paired samples from HSE and HSM patients (*n* = 8/group) were analyzed. Overall, the constitutive levels of chemokines were higher in serum compared to CSF (Table 2, Table 3, Additional file 1: Table S1). CCL15 and CCL21 were both present at ng/ml concentrations in serum of control subjects and an additional 12 chemokines were expressed at high levels (Additional file 1: Table S1). In HSE six chemokines had a moderate but significant increase in serum compared to controls (CCL20, CCL27, CX3CL1, CXCL2, CXCL9, CXCL11), while only one increased in serum of HSM (CCL21) (Additional file 1: Table S1). Among cytokines, only MIF increased in serum, and only in HSM (Additional file 1: Table S1).

Performing Spearman’s rank correlation between serum and CSF levels of the 40 measured analytes, statistical correlations were observed for CXCL9 (*r* = 0.74) and CXCL13 (*r* = 0.76) in HSE and for CCL7 (*r* = 0.83) and CCL24 (*r* = 0.79) in HSM. There was also inverse correlations for CXCL6 (*r* = −0.81), CXCL16 (*r* = −0.86), IL-10 (*r* = −0.74), and IL-16 (*r* = −0.74) in HSE (Table 3). No other correlations were observed (Table 3).

## Discussion

In this study, we demonstrate that CNS disease caused by the closely related HSV-1 and HSV-2 present with different local patterns of chemokine and cytokine expression. Both conditions were associated with high levels of CXCL8, CXCL9, and CXCL10 and increased levels of many cytokines in CSF. However, HSM but not HSE was associated with high levels of CXCL11 in CSF, and these diseases might thus be distinguished through the induction of this chemokine.

Chemokines function as immune cell attractants and can in some cases directly activate leukocytes to produce cytokines [30]. We show that 13 of 30 measured chemokines and one out of 10 measured cytokines are expressed in healthy CSF. Constitutive expression in the CNS of several monocyte-attracting chemokines, like CCL2, CX3CL1, CCL15, and CCL20, has previously been reported [31, 32]. We confirm constitutive expression for all of them except CCL20. CCL21 has been reported to be constitutively expressed in non-lymphoid tissues [33], but to the authors knowledge has previously not been described in the CNS. MIF is the only cytokine in this analysis with known constitutive expression, in contrast to other cytokines that are produced upon immune activation [34]. However, five chemokines had expression in CSF exceeding steady-state levels in serum; CCL2, CXCL8, CXCL10, CXCL12, and CXCL16. We propose that these chemokines are involved in the immune surveillance of CNS during steady state. T cells make up 80% of immune cells in healthy CSF [35], thus could be the main target cell for these chemokines.

We found that few chemokines were upregulated in CSF during HSE or HSM to levels exceeding those in serum. CXCL8 was found in CSF of both HSE and HSM, but the levels were far higher in HSM. High levels of CXCL8 have been reported in the CSF of HSE patients, where levels seem to be stable for a long period after disease onset [36]. CXCL8 binds receptors CXCR1 and CXCR2, which are both found on neutrophils and induce neutrophil migration through CXCR2. However, we observed few polynuclear cells in CSF of both HSE and HSM patients. This indicates that neutrophils enter the CNS upon disease onset and quickly disappear, or that an additional signal is needed for neutrophil migration into the CNS. Since high levels of CXCL8 are observed in both bacterial and viral meningitis, but only bacterial meningitis is accompanied by a potent influx of neutrophils, we propose that this additional signal is missing in HSV CNS inflammations.

The common proinflammatory chemokines CXCL9 and CXCL10 are upregulated in CSF in both HSE and HSM, as in many other inflammatory CNS conditions [30, 37, 38]. High CSF CXCL11 levels on the other hand were exclusive for HSM. All three chemokines bind the chemokine receptor CXCR3, which is mainly found on CD4^+^ and CD8^+^ T cells. The affinity for CXCR3 is highest for CXCL11, followed by CXCL10 and CXCL9 [26]. We suggest that the high levels of CXCL11 could explain the greater influx of mononuclear cells secreting IFN-γ into CSF in HSM. CXCL9, CXCL10, and CXCL11 are induced by IFN-γ, but interestingly CXCL11 is as also induced by IFN-β [26]. This reflects differences in the promotor elements for CXCL9, CXCL10, and CXCL11. For example, the promotors for CXCL10 and CXCL11 both contain IRSE elements whereas the promotor for CXCL9 does not. In addition, a STAT3-STAT1 heterodimer binds to the CXCL11 promotor whereas a STAT1-STAT2 heterodimer binds to the promotors for CXCL9 and CXCL10 [26].

Overall, very little is known about chemokines and their receptor interactions in the human CNS. CXCL10 is known to be expressed in the CNS by microglia, neurons, and stromal cells. Depending on type of CNS infection CXCL10 has been found to have protective as well as detrimental function in disease progression [39]. Mice infected with HSV-2 and deficient in CXCL9 or CXCL10 have higher viral titers and impaired recruitment of NK cells and virus-specific CD8^+^ T cells to the CNS [40]. Susceptibility to HSV-2 increase in CXCR3-deficient mice, and they succumb faster to disease [41]. However, mice infected with HSV-1 and deficient in CXCR3 are protected from fatal CNS disease [42]. The role of CXCR3 and its ligands are likely tissue-specific and dependent on mouse strain [43].

CXCL11 may have a role in tolerance induction by influencing generation of IL-10^hi^Foxp3^-^ T cells with a regulatory phenotype, whereas CXCL10 instead potentiate a strong Th1 polarization [44]. CXCL11 could thus account for a better disease resolution in HSM because of initiation of an anti-inflammatory regulatory T cell response which could prohibit tissue damage. On the other hand, the induction of a tolerogenic state could warrant the recurrent episodes of HSM. CXCL11 expression is not unique for HSM as it has been observed in neuroborreliosis and enteroviral meningitis [45, 46]. However, the levels of CXCL11 were modest compared to HSM.

CCL8 was the only CCL chemokine with a CSF/serum factor above 1, and only for HSM. CCL8 is not a well-studied chemokine, and has mainly been implicated in Th2 responses, but is associated with lymphocyte and monocyte migration as well as stimulation of eosinophils and basophils [47]. Increased CSF levels of CCL8 have been found in undefined viral meningitis, in pneumococcal meningitis, and in neuroborreliosis [38, 48], but not in *Listeria monocytogenes* meningitis [49].

HSM predominantly affects women, which is reflected in the patient group of this study. To rule out that the differences in chemokine levels in the CSF between HSE and HSM were due to sex bias, we performed a subgroup analysis of the female patients which confirmed that the differences in CCL8 and CXCL11 were indeed attributed to their respective disease.

Cytokines were not analyzed using the CSF/serum factor, since they exert their function at the site of inflammation. More cytokines analyzed were enhanced in HSM compared to HSE, which indicate that HSM is associated with a higher CSF influx of cytokine-secreting cells. Most of the measured cytokines are related to pro-inflammatory Th1 response, like IFN-γ, TNF-α, and IL-2 [50], which would suggest that the infiltrating T cells are predominantly of Th1 lineage. However, it is interesting that we find very high levels of IL-6, a cytokine commonly associated with differentiation of Th2 cells and induction of antibody production in B cells [51]. The cytokine response in HSE is generally low and suggests that cytokines either do not reach the CSF, or that the immune system is less responsive to HSV-1 or its location. The production of cytokines confirm that there is an infiltration of Th1 associated cells as well as other blood-derived cells into the CNS in response to chemokine gradients, and that these gradients seems to be stronger in HSM compared to HSE.

We found no evidence of in- or efflux of chemokines/cytokines between CSF and serum of HSE or HSM patients. Correlation data where CSF levels of chemokines were compared to serum chemokine levels argue against leakage of chemokines between these two compartments. In serum, six chemokines were increased in HSE, compared to only one in HSM. Cytokines and chemokines are likely to be locally produced in the CNS during HSE and HSM, and probably both by infiltrating immune cells as well as resident cells like microglia and astrocytes [39, 52].

## Conclusions

The difficulty in treating HSE to avoid neurological complications poses a great challenge. Here, we show that the production of biomolecules in HSE and HSM are different. Despite the fact that HSE is a significantly more severe disease than HSM, a stronger and a more diverse chemokine and cytokine CSF response is observed in HSM, with higher levels of mononuclear cells. Strong induction of CXCL8, CXCL9, and CXCL10 is observed in both diseases, but only HSM is accompanied by high levels of CXCL11 and CCL8. Whether CXCL11 and CCL8 are universal markers of meningitis but not encephalitis, or if HSV-1 and HSV-2 differ in their ability to induce the production of these chemokines, remains to be determined. It is also unclear if the lack of CXCL11 and CCL8 contributes to the disease severity in HSE and/or to relapsing disease in HSM, and should be further studied to potentiate better treatment for both patient groups.

## Additional files

Additional file 1: Table S1. Levels of chemokines and cytokines in serum of HSE and HSM patients and healthy controls. (XLSX 18 kb)

Additional file 2: Figure S1. Levels of (A) CXCL10, (B) CXCL11 and (C) CCL8 in CSF of female HSE (*n* = 6-7) and HSM (*n* = 7-17) patients compared to healthy controls (*n* = 7-15). Data are presented as individual values with medians indicated by horizontal bars. CSF comparisons were performed using Kruskal-Wallis’ non-parametric test with Dunn’s post-test. Statistical analysis was performed using GraphPad Prism version 6 (GraphPad Software). Abbreviations: CSF, cerebrospinal fluid; HSE, herpes simplex encephalitis; HSM, herpes simplex meningitis. (PPTX 1582 kb)

## Abbreviations

BBB: Blood-brain barrier
BCSFB: Blood-cerebrospinal fluid barrier
CNS: Central nervous system
CSF: Cerebrospinal fluid
HSE: Herpes simplex type 1 encephalitis
HSM: Herpes simplex type 2 meningitis
HSV: Herpes simplex virus
HSV-1: Herpes simplex virus type 1
HSV-2: Herpes simplex virus type 2
MRI: Magnetic resonance imaging
IFN: Interferon
MIF: Macrophage migration inhibitory factor
NK: Natural killer
RT: Room temperature

## References

[1] Wald A, Corey L. Persistence in the population: epidemiology, transmission. In: Arvin A, Campadelli-Fiume G, Mocarski E, et al. editors. Human Herpesviruses: Biology, Therapy, and Immunoprophylaxis. Cambridge: Cambridge University Press; 2007. 21348071

[2] Kallio-Laine K (2009). Recurrent lymphocytic meningitis positive for herpes simplex virus type 2. Emerg Infect Dis.

[3] Franzen-Rohl E (2008). High diagnostic yield by CSF-PCR for entero- and herpes simplex viruses and TBEV serology in adults with acute aseptic meningitis in Stockholm. Scand J Infect Dis.

[4] Berger JR, Houff S (2008). Neurological complications of herpes simplex virus type 2 infection. Arch Neurol.

[5] Simko JP (2002). Differences in laboratory findings for cerebrospinal fluid specimens obtained from patients with meningitis or encephalitis due to herpes simplex virus (HSV) documented by detection of HSV DNA. Clin Infect Dis.

[6] Whitley, R.J., Herpes simplex virus, in infections of the central nervous system, W.M. Scheld, R.J. Whitley, and C.M. Marra, Editors. Philadelphia: Lippincott Williams & Wilkins; 2004

[7] Jennische E (2015). The anterior commissure is a pathway for contralateral spread of herpes simplex virus type 1 after olfactory tract infection. J Neurovirol.

[8] Domingues RB (1997). Evaluation of the range of clinical presentations of herpes simplex encephalitis by using polymerase chain reaction assay of cerebrospinal fluid samples. Clin Infect Dis.

[9] Raschilas F (2002). Outcome of and prognostic factors for herpes simplex encephalitis in adult patients: results of a multicenter study. Clin Infect Dis.

[10] Gordon B (1990). Long-term cognitive sequelae of acyclovir-treated herpes simplex encephalitis. Arch Neurol.

[11] Bergstrom T (1990). Primary and recurrent herpes simplex virus type 2-induced meningitis. J Infect Dis.

[12] Aurelius E (2002). Neurologic morbidity after herpes simplex virus type 2 meningitis: a retrospective study of 40 patients. Scand J Infect Dis.

[13] Aurelius E (1994). Cytokines and other markers of intrathecal immune response in patients with herpes simplex encephalitis. J Infect Dis.

[14] Glimaker M, Olcen P, Andersson B (1994). Interferon-gamma in cerebrospinal fluid from patients with viral and bacterial meningitis. Scand J Infect Dis.

[15] Franzen-Rohl E (2011). Increased cell-mediated immune responses in patients with recurrent herpes simplex virus type 2 meningitis. Clin Vaccine Immunol.

[16] Skoldenberg B (1984). Acyclovir versus vidarabine in herpes simplex encephalitis. Randomised multicentre study in consecutive Swedish patients. Lancet.

[17] Studahl M (2013). Acute viral infections of the central nervous system in immunocompetent adults: diagnosis and management. Drugs.

[18] Brigden D (1981). Acyclovir—a review of the preclinical and early clinical data of a new antiherpes drug. Antiviral Res.

[19] Shechter R, London A, Schwartz M (2013). Orchestrated leukocyte recruitment to immune-privileged sites: absolute barriers versus educational gates. Nat Rev Immunol.

[20] Engelhardt B, Coisne C (2011). Fluids and barriers of the CNS establish immune privilege by confining immune surveillance to a two-walled castle moat surrounding the CNS castle. Fluids Barriers CNS.

[21] Ransohoff RM, Engelhardt B (2012). The anatomical and cellular basis of immune surveillance in the central nervous system. Nat Rev Immunol.

[22] Hawkins BT, Davis TP (2005). The blood-brain barrier/neurovascular unit in health and disease. Pharmacol Rev.

[23] Melik-Parsadaniantz S, Rostene W (2008). Chemokines and neuromodulation. J Neuroimmunol.

[24] Griffith JW, Sokol CL, Luster AD (2014). Chemokines and chemokine receptors: positioning cells for host defense and immunity. Annu Rev Immunol.

[25] Muller M (2010). Review: The chemokine receptor CXCR3 and its ligands CXCL9, CXCL10 and CXCL11 in neuroimmunity--a tale of conflict and conundrum. Neuropathol Appl Neurobiol.

[26] Groom JR, Luster AD (2011). CXCR3 ligands: redundant, collaborative and antagonistic functions. Immunol Cell Biol.

[27] McColl SR (2004). Expression of rat I-TAC/CXCL11/SCYA11 during central nervous system inflammation: comparison with other CXCR3 ligands. Lab Invest.

[28] Namvar L (2005). Detection and typing of Herpes Simplex virus (HSV) in mucocutaneous samples by TaqMan PCR targeting a gB segment homologous for HSV types 1 and 2. J Clin Microbiol.

[29] Bremell D (2014). Automated cerebrospinal fluid cell count—new reference ranges and evaluation of its clinical use in central nervous system infections. Clin Biochem.

[30] Melchjorsen J, Sorensen LN, Paludan SR (2003). Expression and function of chemokines during viral infections: from molecular mechanisms to in vivo function. J Leukoc Biol.

[31] Meeker RB (2011). Protein changes in CSF of HIV-infected patients: evidence for loss of neuroprotection. J Neurovirol.

[32] Meeker RB (2012). Cell trafficking through the choroid plexus. Cell Adh Migr.

[33] Lo JC (2003). Differential regulation of CCL21 in lymphoid/nonlymphoid tissues for effectively attracting T cells to peripheral tissues. J Clin Invest.

[34] Lue H (2002). Macrophage migration inhibitory factor (MIF): mechanisms of action and role in disease. Microbes Infect.

[35] Ousman SS, Kubes P (2012). Immune surveillance in the central nervous system. Nat Neurosci.

[36] Rosler A (1998). Time course of chemokines in the cerebrospinal fluid and serum during herpes simplex type 1 encephalitis. J Neurol Sci.

[37] Michlmayr D, Lim JK (2014). Chemokine receptors as important regulators of pathogenesis during arboviral encephalitis. Front Cell Neurosci.

[38] Hickey MJ, Lane TE (2008). The Usual Suspects: Chemokines and Microbial Infection of the Central Nervous System. Central Nervous System Diseases and Inflammation.

[39] Michlmayr D, McKimmie CS (2014). Role of CXCL10 in central nervous system inflammation. Int J Interferon Cytokine Mediat Res.

[40] Thapa M (2008). CXCL9 and CXCL10 expression are critical for control of genital herpes simplex virus type 2 infection through mobilization of HSV-specific CTL and NK cells to the nervous system. J Immunol.

[41] Thapa M, Carr DJ (2009). CXCR3 deficiency increases susceptibility to genital herpes simplex virus type 2 infection: Uncoupling of CD8+ T-cell effector function but not migration. J Virol.

[42] Zimmermann J, et al. Enhanced viral clearance and reduced leukocyte infiltration in experimental herpes encephalitis after intranasal infection of CXCR3-deficient mice. J Neurovirol. 2017;23(3):394–403. 10.1007/s13365-016-0508-6 28116674

[43] Lundberg P (2007). Effects of CXCR3 signaling on development of fatal encephalitis and corneal and periocular skin disease in HSV-infected mice are mouse-strain dependent. Invest Ophthalmol Vis Sci.

[44] Zohar Y (2014). CXCL11-dependent induction of FOXP3-negative regulatory T cells suppresses autoimmune encephalomyelitis. J Clin Invest.

[45] Rupprecht TA (2005). CXCL11 is involved in leucocyte recruitment to the central nervous system in neuroborreliosis. J Neurol.

[46] Cavcic A (2011). Concentration gradient of CXCL10 and CXCL11 between the cerebrospinal fluid and plasma in children with enteroviral aseptic meningitis. Eur J Paediatr Neurol.

[47] Proost P, Wuyts A, Van Damme J (1996). Human monocyte chemotactic proteins-2 and -3: structural and functional comparison with MCP-1. J Leukoc Biol.

[48] Kastenbauer S (2005). Patterns of protein expression in infectious meningitis: a cerebrospinal fluid protein array analysis. J Neuroimmunol.

[49] Koopmans MM (2014). Cerebrospinal fluid inflammatory markers in patients with meningitis. BBA Clin.

[50] Szabo SJ (2003). Molecular mechanisms regulating Th1 immune responses. Annu Rev Immunol.

[51] Diehl S, Rincon M (2002). The two faces of IL-6 on Th1/Th2 differentiation. Mol Immunol.

[52] Semple BD, Kossmann T, Morganti-Kossmann MC (2010). Role of chemokines in CNS health and pathology: a focus on the CCL2/CCR2 and CXCL8/CXCR2 networks. J Cereb Blood Flow Metab.

