# Supplementary figures and images for: CXCL11 production in cerebrospinal fluid distinguishes herpes simplex meningitis from herpes simplex encephalitis
Source: J Neuroinflammation. 2017 Jul 10;14:134. doi: 10.1186/s12974-017-0907-5 (PMC5504603; doi:10.1186/s12974-017-0907-5)

## Slide 1
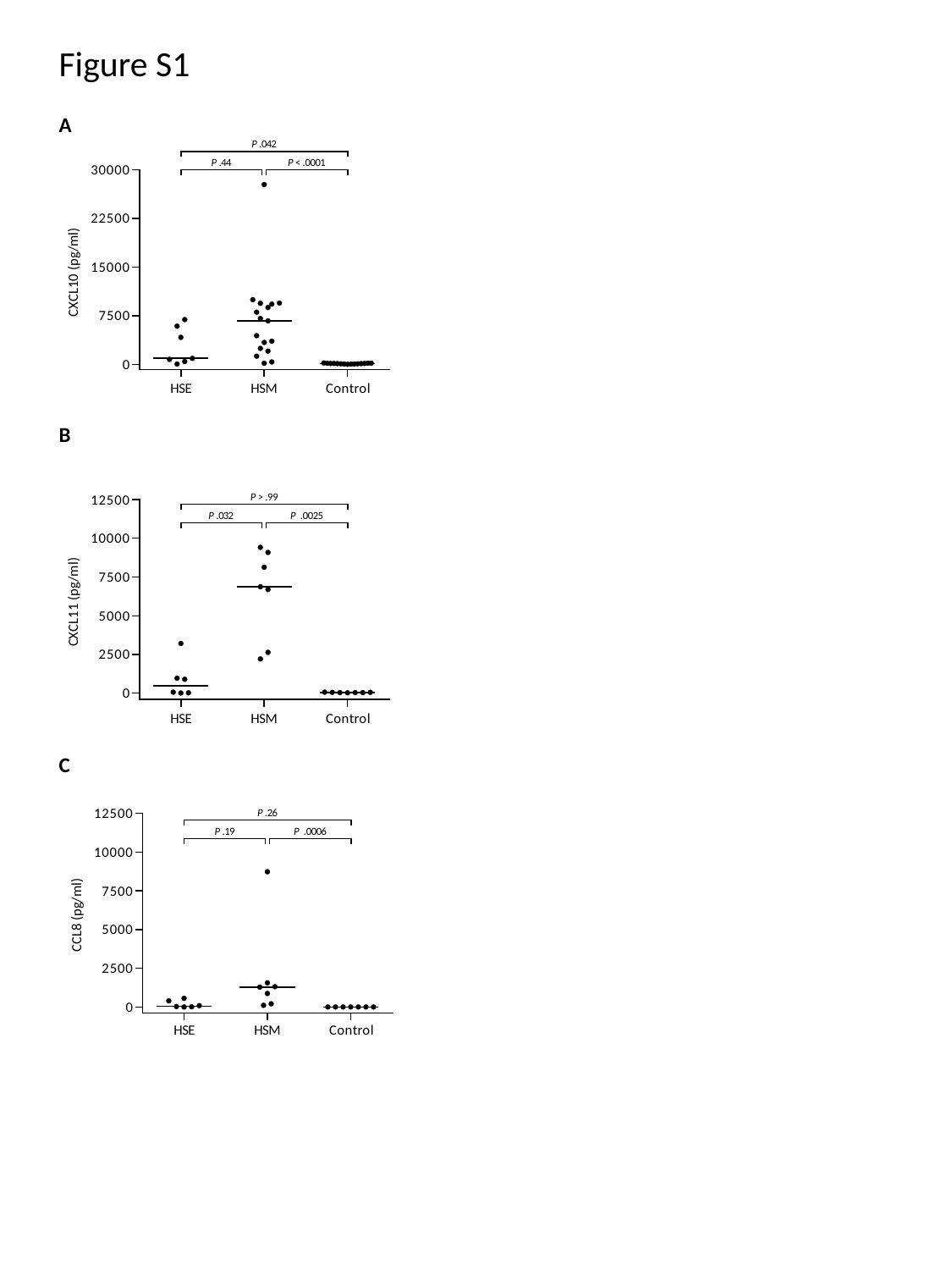

# Figure S1
A
B
C

Supplement: Supplementary file 2 — Levels of (A) CXCL10, (B) CXCL11 and (C) CCL8 in CSF of female HSE (n = 6-7) and HSM (n = 7-17) patients compared to healthy controls (n = 7-15). Data are presented as individual values with medians indicated by horizontal bars. CSF comparisons were performed using Kruskal-Wallis’ non-parametric test with Dunn’s post-test. Statistical analysis was performed using GraphPad Prism version 6 (GraphPad Software). Abbreviations: CSF, cerebrospinal fluid; HSE, herpes simplex encephalitis; HSM, herpes simplex meningitis. (PPTX 1582 kb) [file 12974_2017_907_MOESM2_ESM.pptx]
